# ABCG2 is associated with HER-2 Expression, lymph node metastasis and clinical stage in breast invasive ductal carcinoma

**DOI:** 10.1186/1746-1596-6-90

**Published:** 2011-09-27

**Authors:** Lei Xiang, Peng Su, Shujun Xia, Zhiyan Liu, Yan Wang, Peng Gao, Genyin Zhou

**Affiliations:** 1 Department of Pathology, Shandong University School of Medicine, 44, Wenhua Xi Road, Ji'nan, Shandong Province, P.R.China

**Keywords:** ABCG2(BCRP), Her-2, Lymph node metastasis, Clinical stage, Correlation, Breast invasive ductal cancer, Immunohistochemistry, Tissue microarray

## Abstract

**Background:**

ABCG2 is an ABC transporter. It has been demonstrated that endogenous ABCG2 expression in certain cancers is a possible reflection of the differentiated phenotype of the cell of origin and likely contributes to intrinsic drug resistance. But little is known about the contribution of ABCG2 to the drug resistance and the clinicopathological characteristics in breast cancer. In the present study, we investigated the expression of ABCG2 and the correlations between ABCG2 expression and patients' clinicopathological and biological characteristics.

**Methods:**

Immunohistochemistry was employed on the tissue microarray paraffin sections of surgically removed samples from 196 breast cancer patients with clinicopathological data.

**Results:**

The results showed that ABCG2 was expressed in different intensities and distributions in the tumor cells of the breast invasive ductal carcinoma. A positive stain for ABCG2 was defined as a brown stain observed in the cytoplasm and cytomembrane. A statistically significant correlation was demonstrated between ABCG2 expression and HER-2 expression (p = 0.001), lymph node metastasis (p = 0.049), and clinical stage (p = 0.015) respectively.

**Conclusion:**

ABCG2 correlated with Her-2 expression, lymph node metastasis and clinical stage in breast invasive ductal carcinoma. It could be a novel potential bio-marker which can predict biological behavior, clinical progression, prognosis and chemotherapy effectiveness.

## Background

Breast cancer is the most common carcinoma in females and the second most common cause of cancer related mortality in women [1], with more than 1,000,000 new cases and 370,000 deaths yearly worldwide [2]. Surgery is the mainstay of the treatment of breast cancer. Many patients receive adjuvant (post-operative) therapy, which reduces the risk of loco-regional and distant disease recurrence. Adjuvant treatment options include chemotherapy, radiotherapy, endocrine therapy and biological agents, aiming to provide maximum survival benefit with minimum toxicity [3]. Systemic therapy improves the disease-free survival of those patients, but does not cure patients with advanced or metastatic disease, and fails to benefit the majority of patients with localized breast cancer. Intrinsic resistance to chemotherapy is emerging as a significant cause of treatment failure [4] and the resistance phenotype is often associated with increased expression of ATP-binding cassette (ABC) transporters that mediate energy-dependent transport of substrate drugs out of the cell against a concentration gradient [5]. ABCG2 (ATP-binding cassette sub-family G member 2), or breast cancer resistance protein (BCRP), is an ABC transporter that has been extensively studied. Its overexpression has been demonstrated that endogenous ABCG2 expression in certain cancers is possibly a reflection of differentiated phenotype of cell origin and may contribute to intrinsic drug resistance in vitro. Notably, research into the transporter's role in cancer drug resistance and its development as a therapeutic target in cancer has lagged [6]. The data about the contribution of ABCG2 to drug resistance in breast cancer are scarce [7-9]. Therefore, further studies are needed to explore the expression of ABCG2 in primary breast cancer and its correlation with the clinicopathological and biological characteristics of the breast cancer.

In the present study, the expression of ABCG2 was investigated by immunohistochemistry using tissue microarray according to immunohistochemical phenotypes and the correlationships between ABCG2 expression and the clinicopathological data. Moreover, biological characteristics were discussed. We demonstrated a possibility of its predictive role in chemotherapy in breast cancer.

## Materials and methods

### Patients and Tissue samples

We retrieved tissue samples from patients with breast invasive ductal carcinoma in the Department of Pathology of Qilu Hospital of Shandong University during July 2007 through December 2008. Formalin-fixed and paraffin-embedded tissue specimens from 196 patients with primary breast cancer were included. All archival hematoxylin and eosin (H&E)-stained slides for each patient were reviewed by two pathologists. For the usage of the clinical materials for research purposes, prior patient consent and approval from the Institutional Research Ethics Committee were obtained. All the diagnoses were made following the Pathology and Genetics of Tumors of Breast of the World Health Organization Classification of Tumors [10]. Clinicopathologic classification and staging were determined according to the American Joint Committee on Cancer criteria [11].

The histological grade was assessed using the Nottingham grading system [12], and nuclear grade was evaluated according to the modified Black's nuclear grade [13]. Histological parameters such as histological subtype, nuclear grade and histological grade were evaluated according to H&E-stained slides. Clinical parameters included patients' age, tumor size, lymph node status, clinical stage and biological markers (ER, PR, HER2 and ki67 et al).

### Tissue microarray

For each H&E-stained slide, two representative areas were selected and the corresponding spots were marked on the surface of the paraffin block. Using a tissue microarray punching instrument, the selected areas were punched out and were placed into the recipient block side by side. Each tissue core was 2 mm in diameter and was assigned with a unique tissue microarray location number that was linked to a database containing other clinicopathologic data.

### Immunohistochemistry (IHC)

The streptavidin-peroxidase-biotin (SP) immunohistochemical method was utilized to study the expression of ABCG2 in 196 paraffin-embedded breast tissues.

In brief, paraffin-embedded specimens were cut into 4 μm sections and baked at 60°C for 60 min. The sections were deparaffinized with xylenes and rehydrated. Then sections were submerged into EDTA antigenic retrieval buffer in a pressure cooker for 10 minutes and then cooled at room temperature for 20 minutes. The sections were treated with 3% hydrogen peroxide in methanol to quench the endogenous peroxidase activity, followed by incubation with normal serum to block nonspecific binding. Mouse monoclonal [BXP-21] (1:50; Abcam Company, ab3380, USA) was incubated with the sections overnight at 4°C; the second antibody was from an SP reagent kit (Zhongshan Biotechnology Company, Beijing, China). After washing, the tissue sections were treated with biotinylated anti-mouse secondary antibody, followed by further incubation with streptavidin-horseradish peroxidase complex for 20 mins. Stained with diaminobenzidine (DAB), the sections were counterstained with hematoxylin. For negative controls, the anti-ABCG2 antibody was replaced with PBS.

### Evaluation of Immunohistochemical Staining

The stained slides were reviewed and scored independently by two observers blinded to the patients' information, and the scores were determined by combining the proportion of positively stained tumor cells and the intensity of staining. Tumor cell proportion was scored as follows [14]: 0 (no positive tumor cells); 1 (≤ 30% positive tumor cells); 2 (31-50% positive tumor cells); 3 (51-80% positive tumor cells) and 4 (> 80% positive tumor cells). Staining intensity was graded according to the following criteria: 0 (-, no staining); 1 (+, weak staining = light yellow); 2 (++, moderate staining = yellow brown) and 3 (+++, strong staining = brown). Staining index (SI) was calculated as the product of the staining intensity score and the proportion of positive tumor cells. Using this method of assessment, we evaluated ABCG2 expression in invasive breast cancer cells by determining the SI, with scores of 0,1, 2, 3, 4, 6, 9 or 12. The optimal cutoff value for high and low expression level was identified: an SI score of ≥ 4 was used to define tumors with high ABCG2 expression, and an SI score of ≤ 3 was used to indicate low ABCG2 expression and the SI score of 0 was used to imply negative expression.

### Statistical Analysis

Analyses were performed using the statistical software package SPSS 13.0 (SPSS, Chicago, IL). The chi-square test or Fisher's exact test were used to evaluate the correlation between ABCG2 expression and the clinicopathologic characteristics if appropriate. Bivariate correlations between study variables were calculated by Spearman's rank correlation coefficients. Differences were considered statistically significant for p < 0.05.

## Results

**Figure 1:**
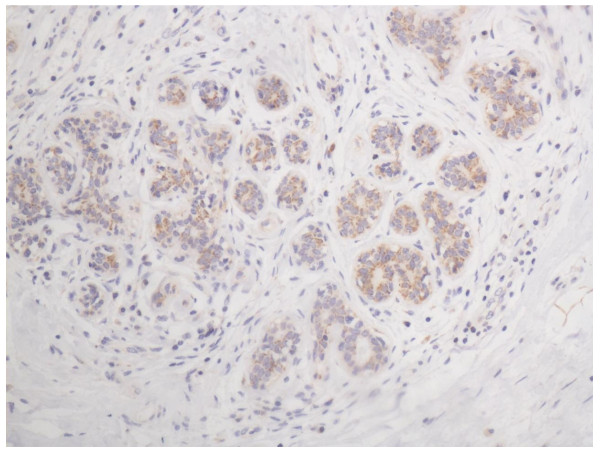
**Normal breast tissue, ABCG2 positive (IHC, SP ×200)**. The ABCG2 staining is weak, and mainly localized in the cytoplasm and cytomembrane of the glandular epithelium cells.

The specificity of the immunodetection was confirmed by using the monoclonal antibodies BXP-21. A positive stain for ABCG2 was defined as a brown stain observed in the cytoplasm and cytomembrane. Positive staining of normal adjacent ductal epithelia as well as vascular endothelium of the breast has a low level expression (Figure 1). And it can serve as an internal positive control. No staining of lymphoid cells was seen.

**Figure 2:**
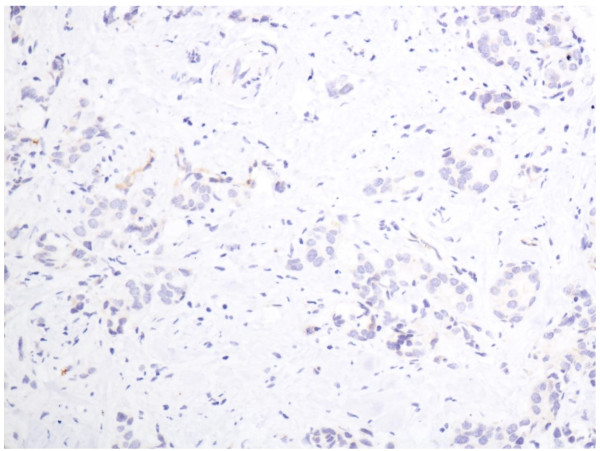
**Breast cancer tissue, ABCG2 negative (IHC, SP ×200)**. The ABCG2 staining is almost negative.

**Figure 3:**
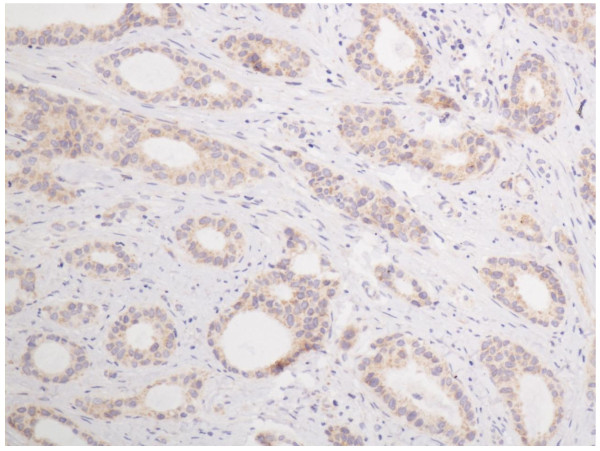
**Breast cancer tissue, ABCG2 positive + (IHC, SP ×200)**. The ABCG2 staining is weak.

**Figure 4:**
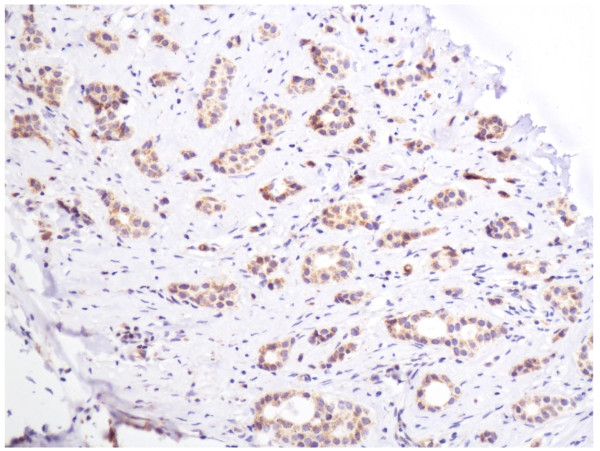
**Breast cancer tissue, ABCG2 positive ++ (IHC, SP ×200)**. The ABCG2 staining is moderate.

**Figure 5:**
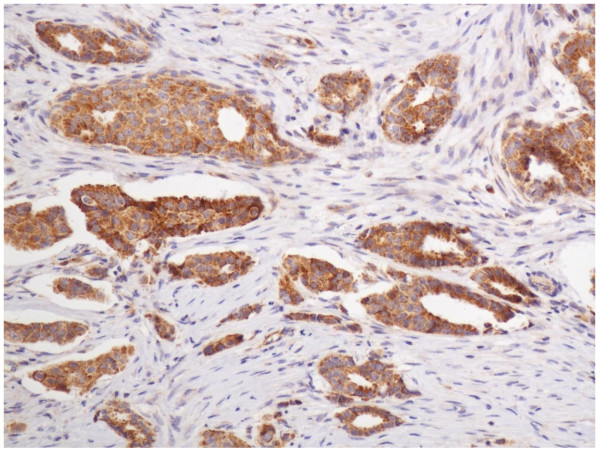
**Breast cancer tissue, ABCG2 positive +++ (IHC, SP ×200)**. The ABCG2 staining is strong.

In the cells of the breast invasive ductal carcinoma, ABCG2 expression was present in different intensities and different cell distributions. Following the staging criteria of stain intensity, 15 cases (7.7%) were identified as completely negative (Figure 2), 80 cases (40.8%) were identified as "+" (Figure 3), 77 cases (39.2%) were identified as "++" (Figure 4), and 24 cases (12.3%) were identified as "+++" (Figure 5). According to the above assessment methods and evaluation criterion, combining the proportion of positively stained tumor cells, the high level, low level and negative expression of ABCG2 was observed in 73 cases (37.2%), 97 cases (49.5%) and 26 cases (13.3%), respectively.

There was no significant correlation between the expression level of ABCG2 and biological factors such as patients' age (p = 0.801), histology type (p = 0.711), histology-grade (p = 0.424), tumour size (p = 0.210), ER (p = 0.136), PR (p = 0.693) and Ki67 (p = 0.906). In contrast, statistical analyses indicated that ABCG2 expression was positively related with HER-2 expression and the correlation was statistically significant (p = 0.001); meanwhile, the correlation between ABCG2 expression and lymph node metastasis/clinical stage was significant (p = 0.049/0.015, respectively). The results of these analyses are summarized in Table 1.

**Table 1:** The correlation between the expression of BCRP and the clinicopathological parameter

	Patients	BCRP expression			P value
			
	n (%)	negative	low	high	
Age					0.801

≤ 50 years	80	10	38	32	

> 50 years	116	16	59	41	

Histology-type					0.711

IDC	155	20	75	60	

IDC with others	41	6	22	13	

Histology-grade					0.424

G1/G2	143	19	67	57	

G3	53	7	30	16	

Tumour size					0.210

≤ 2.0 cm	97	9	48	40	

> 2.0 cm	99	17	49	33	

LNM					0.049*

-	103	8	56	39	

+	93	18	41	34	

ER					0.136

-/+	96	14	53	29	

++/+++	100	12	44	44	

PR					0.693

-/+	128	18	65	45	

++/+++	68	8	32	28	

HER2					0.001*

-/+	138	18	57	63	

++/+++	58	8	40	10	

Ki67					0.906

Positive ≤ 50%	154	21	75	58	

Positive > 50%	42	5	22	15	

Clinical stage					0.015*

Stage I	60	2	32	26	

Stage IIa and IIb	86	11	44	31	

StageIIIa and IIIb	50	13	21	16	

*P *values were evaluated by chi-square test or the Fisher's exact test. *P < 0.05.

## Discussion

ABCG2, or breast cancer resistance protein (BCRP), is the second member of the G subfamily of the ATP-binding cassette (ABC) efflux transporter superfamily that has been the subject of intense study since its discovery two decades ago. In 1998, Doyle et al. cloned the gene responsible for the novel resistance phenotype from the MCF-7 Adr/Vp subline in the absence of P-gp or MRP1. They named the gene BCRP for breast cancer resistance protein since it was cloned from a breast cancer subline. Soon after, two nearly identical transporters, termed ABCP for the ABC transporter highly expressed in placenta [15] and MXR for the mitoxantrone resistance gene[16], were found in different labs. When the sequences for the genes BCRP/ABCP/MXR became available, they proved to be nearly identical. The Human Genome Nomenclature Committee assigned the gene the name ABCG2, making it the second gene in the G subfamily of ABC transporters that is made up of only half-transporters.

The human ABCG2 gene is localized on chromosome 4, band 4q21-4q22 in normal cells [17]. It extends over 66 kb containing 16 exons and 15 introns, and the resulting protein is 655 amino acids long [18]. The development of antibodies to ABCG2 enabled the detection of ABCG2 in formalin-fixed, paraffin-embedded tissues. Maliepaard et al. examined ABCG2 expression in normal tissues using the BXP-21 and BXP-34 monoclonal antibodies. ABCG2 expression was found in placenta, particularly in the synctiotrophoblastic cells, as well as in colon, small intestine, biliary canaliculi, breast tissue, venous endothelium, and in capillaries [19].

In the present study, we studied ABCG2 expression in paraffin-embedded tumor samples using the IHC method with the BXP-21 antibody, and the positive stain for ABCG2 is located in the cytoplasm and cytomembrane of cells as the product leaflet of the antibody indicates. The expression of ABCG2 in the normal mammary glandular epithelia and in the stromal cells is consistent with previous literature.

In solid tumors, Diestra et al. studied ABCG2 expression in paraffin-embedded tumor samples with the BXP-21 antibody and reported frequent expression in tumors of the digestive tract, endometrium, lung and melanoma [20]. Breast cancer has been most extensively studied, with most reports concluding that ABCG2 expression was relatively low and did not appear to correlate with clinical outcome in the studies of Kanzak et al. [7] or Faneyte et al. [8].

Inconsistent with the above reports, in our study, the expression of ABCG2 in most tumor cells (86.7%) of the breast invasive ductal carcinoma is positive, in which about 40% cases present relatively high ABCG2 expression in the breast cancer cells. Furthermore, the expression of ABCG2 is correlated with the expression of HER2 significantly (p = 0.001) by means of statistic analysis of 196 breast cancer cases detected by IHC. Those patients with high expression of ABCG2 were demonstrated to be more frequently showing high immunoreactions with HER2, which suggests that ABCG2 may have some correlation with worse biological behavior and clinical aggressiveness. Besides, the expression of ABCG2 is correlated with lymph node metastasis (p = 0.049) and clinical stage (p = 0.015), which suggests that the patients with high expression of ABCG2 may have a worse prognosis than those with low expression of ABCG2.

In the previous reports, much fewer cases (43 cases and 52 cases, respectively) were examined than that in the present study (196 cases). They mainly detected the levels of BCRP mRNA in those cases, and only 27 cases of breast cancer underwent IHC testing to examine the expression of BCRP protein [7,8]. These may explain the differences between their studies and the present one. To the best of our knowledge, this is the first report that the expression of ABCG2 is correlated with some clinicopathological parameters and biological markers in the cohort of breast invasive ductual carcinoma. We think it is of great importance for further research.

As we all know, HER2 is an oncogene that has been studied extensively. Human epidermal growth factor receptor 2 (ERBB2, formerly HER2/neu, c-erbB2), 1 of a family of 4 membrane tyrosine kinases (TKs), was found to be amplified in a human breast cancer cell line 26 years ago [21], and this amplification was shown to be important in the pathogenesis and progression of human breast cancer [22]. Since that time, HER2 amplification and resultant HER2 protein overexpression have been linked to important tumor cell proliferation and survival pathways, several drugs have been developed to target the pathway, and the detection of HER2 has become a routine prognostic and predictive factor in breast cancer[23].

Normal tissues have a low complement of HER2 membrane protein. Overexpression of HER2 is seen in 20% of breast cancers, and it confers worse biological behavior and clinical aggressiveness in breast cancer [22,24]. The differential in HER2 expression between normal tissues and tumors helps to define HER2 as an ideal treatment target. Trastuzumab, the first treatment targeting HER2, is well tolerated in patients and has little toxicity because its effects are relatively specific for cancer cells over expressing HER2.

Thus, the significant correlation (p = 0.001) between the expression of ABCG2 and of HER2 may suggest that ABCG2 is not only an ABC transporter which plays a role in drug-resistance of breast cancer chemotherapy but also a novel potential bio-marker which can predict biological behavior, clinical progression, prognosis and chemotherapy effectiveness. And it indicates that when the tumors are treated with chemotherapy and targeting medicines, the antagonists of ABCG2 are supposed to be applied together.

Because the activated AKT mediates the metastasis of tumor cells by the HER2/PI3K/PTEN/AKT pathway [25], HER2 has direct correlation with lymph node metastasis of breast cancer. Due to the strong correlation between the expression of ABCG2 and of HER2, it is easy to understand the correlation between ABCG2 and lymph node metastasis status (p = 0.049) found in this study. Clinical stage is dependent on the lymph node metastasis in large extent, and the correlation between them is obvious. So the significant correlation (p = 0.015) between the expression of ABCG2 and clinical stage found in the present study is pleasant to be accepted by us. Because there was no case in clinical IV stage in the 196 patients which were used in the study, it is not possible to evaluate the correlation between ABCG2 expression and cases with distant metastasis.

Two functional elements in the ABCG2 promoter, the estrogen [26] and hypoxia [27] response elements, and a peroxisome proliferator-activated receptor g (PPARg) response element upstream of the ABCG2 gene [28] have been shown to control ABCG2 expression. So it seems that the expression of ER should be correlated with ABCG2, but the result of our study failed to show this. It is not disappointing, because previous studies have showed that the effects of E2 on ABCG2 expression in breast cancer cells were inconsistent and tissue-specific as a result of the differences between ERαand ERβ. In several human breast cancer cell lines, E2 exposure decreases BCRP protein expression and function, but it does this by acting through ERαand not ERβ[29]. However, E2 has also been reported to increase BCRP protein expression in a human breast cancer cell line by signaling through ERα[30]. In a human placenta cell line, E2 signaled through ERβ to up-regulate BCRP [31]; and Anika M S Hartz et al. found that E2 signals through ERβ, PTEN/PI3K/Akt/GSK3 to down-regulate the expression of BCRP [32]. Thus, both ERαand ERβ can be involved in E2 regulation of BCRP, but the signals involved and the effect on BCRP (up- or down-regulation) seem to be inconsistent and tissue-specific. These may be the reason why the correlation between ABCG2 and ER is not significant (p = 0.136).

As to the possible regulation signaling pathway between HER2 and ABCG2, we speculate some possibilities below. A recent study by Bleau et al. demonstrated that PTEN/PI3K/Akt signaling regulates ABCG2 activity in mouse and human gliomas [33], and PTEN/PI3K/AKT pathway has already been confirmed be one of the signal-transmitting pathways in the HER signaling network. HER2 partners with HER3 to form heterodimerization, and HER3 has multiple docking sites for PI3K. So, HER2 partnering with HER3 is the most potent stimulator of the PI3K/AKT pathway [27].

The HER2/PTEN/PI3K/AKT/ABCG2 pathway may be the signaling regulation mechanism between HER2 and ABCG2. Of course, all the above speculated regulation pathways need to be certificated by further study.

## Conclusion

Our study demonstrates the expression of ABCG2 in normal ductal epithelia and in the tumor cells of invasive ductal carcinoma of breast. Its expression is correlated significantly with that of HER2, lymph node metastasis, and clinical stage in invasive ductal breast cancer. These findings suggest that ABCG2 not only is one of the drug-resistance genes, but also is a novel potential bio-marker which can predict biological behavior, clinical progression, prognosis and chemotherapy effectiveness. Further investigation on the molecular mechanism of possible regulation relationships between them is warranted, especially on the PI3K/Akt signaling pathway.

## Competing interests

The authors declare that they have no competing interests.

## Authors' contributions

LX and YW did the immunohistochemical analysis. PS and SX reviewed all the pathological slides and made the tissue microarray. ZL analyzed the data. GZ designed the study. All authors read and approved the final manuscript.
